# Renal Function and Body Mass Index Contribute to Serum Neurofilament Light Chain Levels in Elderly Patients With Atrial Fibrillation

**DOI:** 10.3389/fnins.2022.819010

**Published:** 2022-04-14

**Authors:** Alexandros A. Polymeris, Fabrice Helfenstein, Pascal Benkert, Stefanie Aeschbacher, David Leppert, Michael Coslovsky, Eline Willemse, Sabine Schaedelin, Manuel R. Blum, Nicolas Rodondi, Tobias Reichlin, Giorgio Moschovitis, Jens Wuerfel, Gian Marco De Marchis, Stefan T. Engelter, Philippe A. Lyrer, David Conen, Michael Kühne, Stefan Osswald, Leo H. Bonati, Jens Kuhle, the Swiss-AF Investigators

**Affiliations:** ^1^Department of Neurology and Stroke Center, University Hospital Basel and University of Basel, Basel, Switzerland; ^2^Department of Clinical Research, University Hospital Basel and University of Basel, Basel, Switzerland; ^3^Cardiovascular Research Institute Basel (CRIB), Basel, Switzerland; ^4^Cardiology Division, Department of Medicine, University Hospital Basel, Basel, Switzerland; ^5^Department of Neurology, Multiple Sclerosis Center and Research Center for Clinical Neuroimmunology and Neuroscience Basel (RC2NB), University Hospital Basel and University of Basel, Basel, Switzerland; ^6^Institute of Primary Health Care (BIHAM), University of Bern, Bern, Switzerland; ^7^Department of General Internal Medicine, University Hospital of Bern, University of Bern, Bern, Switzerland; ^8^Department of Cardiology, University Hospital of Bern, University of Bern, Bern, Switzerland; ^9^Cardiology Division, Department of Medicine, Ente Ospedaliero Cantonale (EOC), Regional Hospital of Lugano, Lugano, Switzerland; ^10^Department of Biomedical Engineering, Medical Image Analysis Center (MIAC) AG, University of Basel, Basel, Switzerland; ^11^Neurology and Neurorehabilitation, University Department of Geriatric Medicine FELIX PLATTER, University of Basel, Basel, Switzerland; ^12^Population Health Research Institute, McMaster University, Hamilton, ON, Canada

**Keywords:** neurofilament light, renal function, glomerular filtration rate, body mass index, elderly, atrial fibrillation

## Abstract

**Objective:**

Serum neurofilament light chain (sNfL) is increasingly used as a neuroaxonal injury biomarker in the elderly. Besides age, little is known about how other physiological factors like renal function and body mass index (BMI) alter its levels. Here, we investigated the association of estimated glomerular filtration rate (eGFR) and BMI with sNfL in a large sample of elderly patients with atrial fibrillation (AF).

**Methods:**

This is a cross-sectional analysis from the Swiss-AF Cohort (NCT02105844). We measured sNfL using an ultrasensitive single-molecule array assay. We calculated eGFR using the chronic kidney disease epidemiology collaboration (CKD-EPI) creatinine (eGFR_crea_) and creatinine–cystatin C (eGFR_crea–cys_) formulas, and BMI from weight and height measurements. We evaluated the role of eGFR and BMI as determinants of sNfL levels using multivariable linear regression and the adjusted R^2^ (R^2^_adj_).

**Results:**

Among 2,277 Swiss-AF participants (mean age 73.3 years), eGFR_crea_ showed an inverse curvilinear association with sNfL after adjustment for age and cardiovascular comorbidities. BMI also showed an independent, inverse linear association with sNfL. The R^2^_adj_ of models with age, eGFR_crea_, and BMI alone was 0.26, 0.35, and 0.02, respectively. A model with age and eGFR_crea_ combined explained 45% of the sNfL variance. Sensitivity analyses (i) further adjusting for vascular brain lesions (*N* = 1,402 participants with MRI) and (ii) using eGFR_crea–cys_ yielded consistent results.

**Interpretation:**

In an elderly AF cohort, both renal function and BMI were associated with sNfL, but only renal function explained a substantial proportion of the sNfL variance. This should be taken into account when using sNfL in elderly patients or patients with cardiovascular disease.

## Introduction

Neurofilament light chain (NfL) is a cytoskeletal protein exclusive to neurons. Following neuroaxonal damage, it is released into the extracellular space, cerebrospinal fluid, and eventually peripheral blood. Over the past years, NfL has been established as the first blood-based biomarker reflecting disease activity and treatment response in traumatic brain injury and neurodegenerative diseases (Khalil et al., 2018; Barro et al., 2020; Gafson et al., 2020). Considering the increasing use of blood NfL as a biomarker for neurological diseases in clinical research and the perspective of its diagnostic and prognostic applications in individual clinical practice, a deeper understanding of its homeostasis (including distribution and clearance) in the blood compartment is needed to elucidate physiological factors that might affect its association with disease processes (Barro et al., 2020; Gafson et al., 2020). This is becoming increasingly important for NfL-based investigations of normal aging (Khalil et al., 2020), as well as cerebrovascular disease (Gattringer et al., 2017; Duering et al., 2018; Tiedt et al., 2018; Peters et al., 2020), atrial fibrillation (AF) (Polymeris et al., 2020), and dementia (Zhao et al., 2019), where accumulating age-related comorbidities might both interfere with the homeostasis of NfL and directly induce neuronal damage *per se* (Barro et al., 2020; Gafson et al., 2020).

While the association of NfL blood levels with age has been consistently demonstrated across a variety of patient populations and healthy controls (Khalil et al., 2018, 2020), their association with renal function and body mass index (BMI) was only recently reported in elderly diabetic patients and younger patients with multiple sclerosis, respectively (Korley et al., 2019; Akamine et al., 2020; Manouchehrinia et al., 2020). However, little is known on how these factors impact NfL concentrations relative to age, one another, and cardiovascular comorbidities and vascular brain lesions, which are increasingly prevalent in the elderly (Vermeer et al., 2007; Wardlaw et al., 2013). Such data are necessary for a systematic appraisal of the importance of these factors as potential confounders and the need to account for them in future use of NfL as a laboratory measure in elderly individuals.

With this in mind, we investigated the association of (i) estimated glomerular filtration rate (eGFR) and (ii) BMI with serum NfL (sNfL) concentrations in a large, well-characterized cohort of elderly AF patients accounting for age, cardiovascular comorbidities, as well as vascular brain lesions and brain volume on neuroimaging.

## Materials and Methods

### Study Design, Patient Population, and Data Collection

This was a cross-sectional analysis using baseline data from the prospective observational Swiss-AF cohort study (NCT02105844), which was designed to investigate the relationship between AF, structural brain changes, and cognition. We selected the Swiss-AF cohort for this analysis due to the large sample size, the detailed clinical and neuroimaging characterization with a relatively high prevalence of cardiovascular comorbidities, and the availability of blood biomarker measurements. Swiss-AF enrolled 2,415 patients with AF between 2014 and 2017 across 14 centers in Switzerland. Included were patients aged 65 years or older, with an additional 15% of patients aged < 65 years. Patients with a recent ischemic stroke, transient ischemic attack (TIA) or other acute illness (< 4 weeks), and those unable to provide consent (e.g., patients with dementia) were excluded. The detailed methodology of Swiss-AF has been described previously (Conen et al., 2017, 2019; Polymeris et al., 2020). Baseline investigations included a standardized clinical assessment (sociodemographic parameters, comorbidities), weight and height measurements [from which BMI was calculated as (weight in kg)/(height in m)^2^], blood sampling, and brain MRI.

Baseline blood samples were collected following standard operating procedures. After centrifugation, serum samples were aliquoted into cryotubes and stored at -80°C in a centralized biobank. The concentration of sNfL was measured in duplicate using a previously described ultrasensitive single-molecule array assay (lower limit of quantification 1.0 pg/ml) (Disanto et al., 2017; Polymeris et al., 2020). Creatinine and cystatin C were measured using commercially available assays (cobas c 311 and Elecsys; Roche Diagnostics, Mannheim, Germany). In order to calculate eGFR as a measure of renal function, we used the Chronic Kidney Disease Epidemiology Collaboration (CKD-EPI) (i) creatinine equation (eGFR_crea_) and (ii) the combined creatinine–cystatin C equation (eGFR_crea–cys_) (Inker et al., 2012).

On baseline MRI, we assessed the presence and volume of small non-cortical infarcts (SNCIs), large non-cortical or cortical infarcts (LNCCIs), and white matter lesions (WMLs); the presence and count of microbleeds (MBs); and estimated the normalized brain volume (nBV) using SIENAX (Smith et al., 2002), as described previously in detail (Conen et al., 2019; Polymeris et al., 2020).

In this study, we included all Swiss-AF patients with quantifiable sNfL measurement and available data on clinical variables, creatinine and cystatin C (Supplementary Figure 1). The Ethics Committee of Northwest and Central Switzerland approved Swiss-AF, including this study (PB_2016-00793). Written informed consent was obtained from all study participants according to the Declaration of Helsinki. This study was conducted in accordance with the STROBE Statement for cross-sectional studies (von Elm et al., 2007).

### Statistical Analyses

#### Main Analysis

As the first step to investigate the association of eGFR and BMI with sNfL, we fitted a multivariable linear regression model with log-transformed sNfL as the dependent variable and eGFR and BMI as independent variables. The model was adjusted for the following variables, known to be associated with sNfL from our previous work in this cohort (Polymeris et al., 2020): age, history of hypertension, diabetes mellitus, stroke or TIA, peripheral artery disease, heart failure, as well as mean arterial pressure [calculated as (1/3 × systolic blood pressure) + (2/3 × diastolic blood pressure)], smoking status (non, past, and current smoker), and alcohol consumption (in standard drinks per day). Continuous variables were centered on their mean (or, in case of skewed data, median) values, as appropriate. Visual inspection suggested curvilinear associations of age and eGFR with sNfL. We chose the best way to model these variables by fitting different univariable models (linear only, quadratic, cubic, cubic without quadratic term) and selecting the one with the best fit based on the Akaike’s information criterion (AIC). Considering the known strong association of age and diabetes with sNfL (Polymeris et al., 2020), we also included in the multivariable model the interactions GFR by age, BMI by age, and GFR by diabetes. We report the backtransformed model-based estimates, which represent multiplicative effects on the geometric mean of sNfL and are denoted by β_*mult*_ (so that a one-unit increase in the independent variable is associated with an average β_*mult*_-fold change in sNfL), along with 95% confidence intervals (95% CI) and two-sided *p*-values.

In a second step, to investigate the relative contribution of eGFR, BMI, and age to the variance of sNfL concentrations, we fitted linear models with log-sNfL as the dependent variable and these factors as independent variables, alone and in combination with one another and with their interactions with age. We report the coefficient of determination (R^2^) and the adjusted R^2^ (R^2^_adj_; penalized for larger number of independent variables) as a measure of the proportion of the observed sNfL variance explained by each model.

#### Sensitivity Analyses

Considering patients who also had baseline brain MRI available (Supplementary Figure 1), we further adjusted the multivariable linear model from the main analysis for the following imaging measures: log-volume of WMLs, presence and log-volume of LNCCIs, presence and log-volume of SNCIs, presence and count of MBs, and nBV, which were previously reported to be associated with sNfL concentrations (Polymeris et al., 2020). We reduced the model to a smaller set of variables via stepwise backward elimination based on AIC. As this is known to inflate the type I error, we refrained from providing *p*-values in this analysis and evaluated the explanatory importance of independent variables for sNfL concentrations based on whether they were selected or eliminated from the reduced model. Finally, we refitted the model without eGFR and report the R^2^ and R^2^_adj_ for both models. We repeated all models using eGFR_crea–cys_ as a further sensitivity analysis.

All analyses were performed with R version 4.0.3 (2020-10-10).

## Results

### Main Analysis

A total of 2,277 Swiss-AF patients were available for the main analysis, after exclusion of 77 patients without sNfL measurement (no or insufficient blood sample), 16 with sNfL measurement below the limit of quantification, and 45 with other data missing (Supplementary Figure 1). The mean [standard deviation (SD)] age was 73.3 (8.5) years, mean (SD) eGFR_crea_/eGFR_crea–cys_ was 59.1 (18.3)/58.6 (20.0) ml/min/1.73 m^2^, mean (SD) BMI was 27.6 (4.7) kg/m^2^, and the median (interquartile range) sNfL was 42.0 (29.0 – 65.1) pg/ml. All patient characteristics are provided in Table 1.

**TABLE 1:** Patient characteristics.

Clinical characteristics of 2,277 Swiss-AF patients (main analysis)	
Age, years, mean (SD)	73.3 (8.5)
Sex, female, N (%)	615 (27.0)
History of atrial fibrillation, N (%)	2,277 (100.0)
History of hypertension, N (%)	1,599 (70.2)
History of diabetes mellitus, N (%)	395 (17.3)
History of stroke or transient ischemic attack, N (%)	452 (19.9)
History of peripheral artery disease, N (%)	183 (8.0)
History of heart failure, N (%)	604 (26.5)
**Smoking status, N (%)**	
Non-smoker	999 (43.9)
Past smoker	1,111 (48.8)
Current smoker	167 (7.3)
Alcohol consumption, std. drinks/day, median (IQR)	0.5 (0.1–1.3)
Mean arterial pressure, mmHg, mean (SD)	92.6 (12.6)
Body mass index, kg/m^2^, mean (SD)	27.6 (4.7)
eGFR_crea_, ml/min/1.73 m^2^, mean (SD)	59.1 (18.3)
eGFR_crea–cys_, ml/min/1.73 m^2^, mean (SD)	58.6 (20.0)
Serum neurofilament light chain, pg/ml, mean (SD)	42.0 (29.0–65.1)

**MRI characteristics of 1,402 Swiss-AF patients (sensitivity analysis)**	

Small non-cortical infarcts, N (%) Volume (if present), mm^3^, median (IQR)	308 (22.0) 62 (30–150)
Large non-cortical and cortical infarcts, N (%) Volume (if present), mm^3^, median (IQR)	299 (21.3) 1,350 (252–7,086)
White matter lesions, N (%) Volume (if present), mm^3^, median (IQR)	1,390 (99.1) 3,753 (1,368–9,353)
Microbleeds, N (%) Count (if present), median (IQR)	302 (21.5) 1 (1–2)
Normalized brain volume, cm^3^, mean (SD)	1,416 (94)

*SD: standard deviation, IQR: interquartile range, eGFR_crea_/eGFR_crea–cys_: estimated glomerular filtration rate based on creatinine/creatinine–cystatin C.*

**FIGURE 1:**
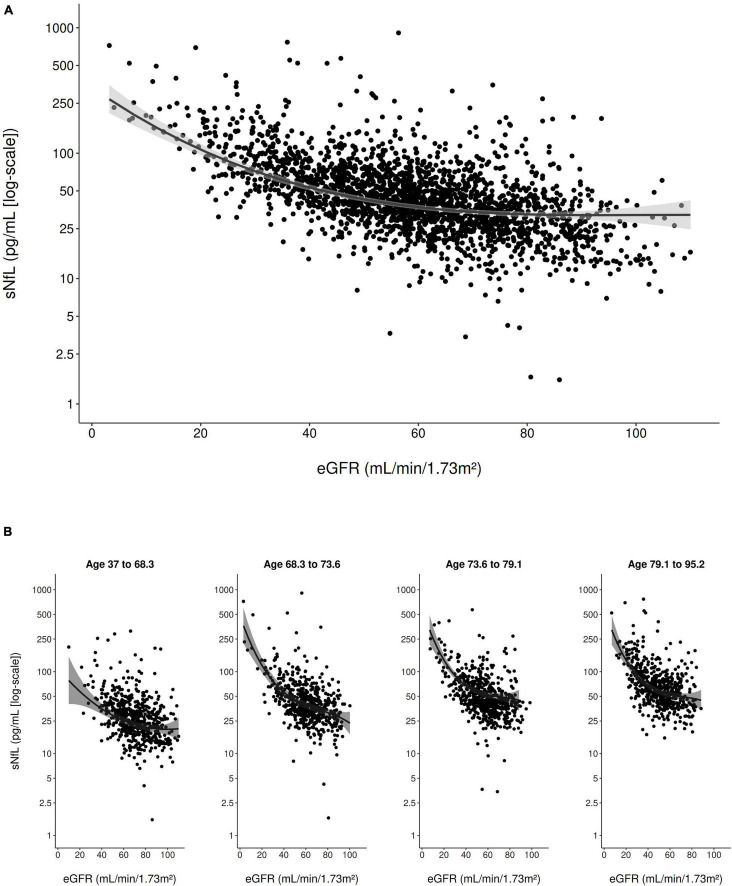
Scatter plot of the association of eGFR_crea_ with sNfL (using the log scale) in the entire study population **(A)** and stratified to age quartiles **(B)**. The solid line represents the predicted values from the main multivariable model and the gray shading represents the 95% confidence interval.

**FIGURE 2:**
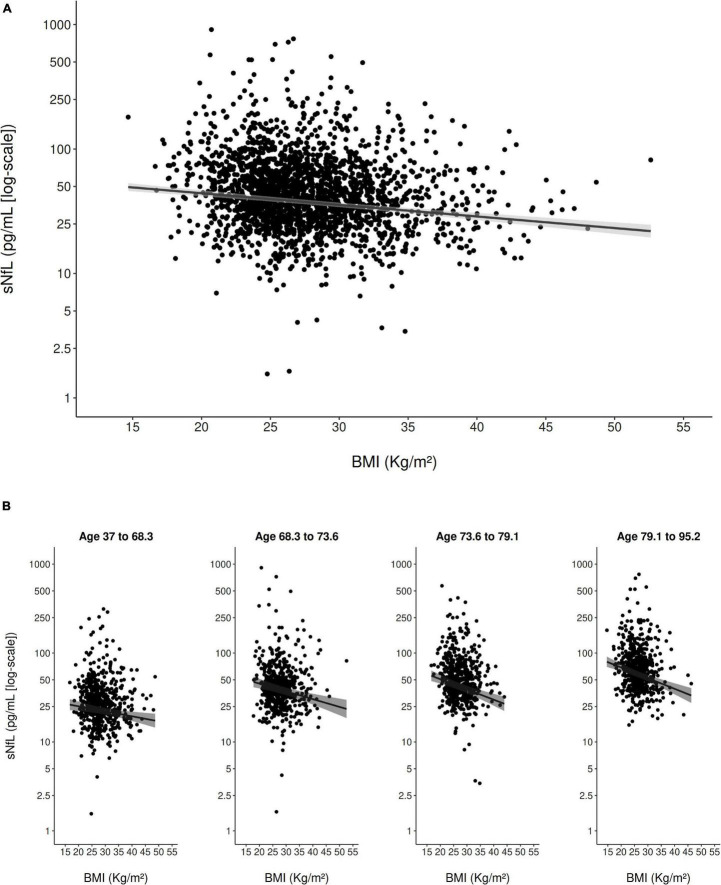
Scatter plot of the association of BMI with sNfL (using the log scale) in the entire study population **(A)** and stratified to age quartiles **(B)**. The solid line represents the predicted values from the main multivariable model and the gray shading represents the 95% confidence interval.

In the multivariable model adjusted for all clinical variables (Table 2), eGFR_crea_ showed a strong inverse curvilinear association with sNfL (Figure 1A). Modeling eGFR_crea_ with a linear, quadratic, and cubic component was chosen based on AIC (Supplementary Table 1). BMI also showed a strong, inverse linear association with sNfL in the multivariable model (Figure 2A).

**TABLE 2:** Multivariable models for the association of eGFR and BMI with sNfL.

Variables (*N* = 2,277)	Using eGFR_crea_			Using eGFR_crea–cys_		
	β_*mult*_	95%-CI	*p*-value	β_*mult*_	95%-CI	*p*-value
Age* (per decade)	1.293	[1.244, 1.344]	< 0.001	1.229	[1.182, 1.278]	< 0.001
[Age* (per decade)]^3^	0.988	[0.977, 0.999]	0.032	0.989	[0.978, 1.000]	0.046
eGFR* (per 10 ml/min/1.73 m^2^)	0.888	[0.869, 0.907]	< 0.001	0.869	[0.854, 0.886]	< 0.001
[eGFR* (per 10 ml/min/1.73 m^2^)]^2^	1.030	[1.023, 1.036]	< 0.001	1.029	[1.024, 1.033]	< 0.001
[eGFR* (per 10 ml/min/1.73 m^2^)]^3^	0.998	[0.996, 1.000]	0.017	0.998	[0.997, 1.000]	< 0.001
BMI* (per 5 kg/m^2^)	0.898	[0.878, 0.919]	< 0.001	0.891	[0.871, 0.910]	< 0.001
History of hypertension	1.043	[0.996, 1.094]	0.076	1.031	[0.985, 1.079]	0.195
History of diabetes mellitus	1.203	[1.137, 1.274]	< 0.001	1.181	[1.117, 1.249]	< 0.001
History of stroke or TIA	1.127	[1.072, 1.185]	< 0.001	1.120	[1.067, 1.176]	< 0.001
History of peripheral artery disease	1.071	[0.993, 1.154]	0.075	1.046	[0.972, 1.125]	0.229
History of heart failure	1.063	[1.014, 1.115]	0.012	1.023	[0.976, 1.071]	0.344
Mean arterial pressure (per 1 mmHg)	0.998	[0.997, 1.000]	0.031	0.999	[0.998, 1.001]	0.277
Past smoker (ref: non-smoker)	0.970	[0.930, 1.011]	0.151	0.967	[0.928, 1.007]	0.109
Current smoker (ref: non-smoker)	0.963	[0.887, 1.044]	0.357	0.934	[0.863, 1.010]	0.088
Alcohol consumption (per 1 std. drink/d)	1.009	[0.988, 1.015]	0.795	1.004	[0.991, 1.017]	0.555
Interaction eGFR × age	1.032	[1.015, 1.050]	< 0.001	1.035	[1.020, 1.049]	< 0.001
Interaction BMI × age	0.971	[0.948, 0.994]	0.013	0.978	[0.956, 1.001]	0.057
Interaction eGFR × diabetes	0.993	[0.967, 1.020]	0.603	1.004	[0.980, 1.029]	0.738

*eGFR: estimated glomerular filtration rate, BMI: body mass index, TIA: transient ischemic attack.*

*Centered on its mean.

Furthermore, age (modeled with a linear and cubic component based on AIC; Supplementary Table 1) was strongly, positively associated with sNfL in the multivariable model. There was evidence for an interaction between eGFR_crea_ and age on their association with sNfL (*p*_*interaction*_ < 0.001), indicating that, with older age, the negative association of eGFR_crea_ with sNfL was steeper for lower values of eGFR_crea_ (Figure 1B). There was also evidence for a weaker interaction between BMI and age on their association with sNfL (*p*_*interaction*_ = 0.013), indicating a slightly stronger negative association of BMI with sNfL with increasing age (Figure 2B). Supplementary Table 2 presents the model-based estimates for the association of eGFR_crea_ and BMI with sNfL in four age-quartile subgroups.

Further variables with a strong association with sNfL in the multivariable model were diabetes mellitus and history of stroke or TIA. There was no evidence for an interaction between eGFR_crea_ and diabetes mellitus on their association with sNfL.

Upon examination of the R^2^_adj_ of different models fitted with sNfL as the dependent variable, models containing age, eGFR_crea_, and BMI alone explained 26%, 35%, and 2% of the variance in sNfL concentrations, respectively. Adding eGFR_crea_ to the age model conferred a substantial increase in the sNfL variance explained by the model (R^2^_adj_ 0.45 vs. 0.26), while adding BMI to age increased the model’s explanatory power only marginally (R^2^_adj_ 0.27 vs. 0.26). The combined age, eGFR_crea_, and BMI model explained 46% of the total sNfL variance. Adding the interaction terms eGFR_crea_ and BMI by age to the models conferred no substantial increase in R^2^_adj_ (Table 3).

**TABLE 3:** Performance of different models including age, eGFR, and BMI to predict serum neurofilament light concentrations.

	Model (*N* = 2,277)								
	Age* alone	eGFR^†^ alone	BMI alone	Age* and eGFR^†^	Age* and eGFR^†^ incl. interaction eGFR × age	Age* and BMI	Age* and BMI incl. interaction BMI × age	Age*, eGFR^†^, and BMI	Age*, eGFR^†^, and BMI incl. interactions eGFR × age, BMI × age
**Using eGFR_crea_**									
R^2^	0.26	0.35	0.02	0.45	0.45	0.27	0.27	0.46	0.47
R^2^_adj_	0.26	0.35	0.02	0.45	0.45	0.27	0.27	0.46	0.46
AIC	3,927	3,629	4,568	3,284	3,269	3,911	3,910	3,231	3,208
**Using eGFR_crea–cys_**									
R^2^	0.26	0.42	0.02	0.48	0.48	0.27	0.27	0.50	0.50
R^2^_adj_	0.26	0.42	0.02	0.48	0.48	0.27	0.27	0.49	0.50
AIC	3,927	3,386	4,568	3,148	3,129	3,911	3,910	3,073	3,044

*R^2^: coefficient of determination, R^2^_adj_: adjusted R^2^, AIC: Akaike’s information criterion.*

*Modeled with a linear and cubic component.

*^†^Modeled with a linear, quadratic, and cubic component.*

### Sensitivity Analysis Adjusting for MRI Variables

A total of 1,402 Swiss-AF patients were available for the MRI sensitivity analysis (Supplementary Figure 1). In the multivariable model including all variables from the main analysis, vascular brain lesions and nBV, both eGFR_crea_ and BMI remained in the model after stepwise backward elimination, as did age, its interaction with eGFR_crea_, diabetes mellitus, and history of stroke or TIA (Supplementary Table 3). The R^2^_adj_ of this model was 52%, and dropped to 36% after excluding eGFR_crea_.

### Sensitivity Analysis Using eGFR_crea–cys_

As for the main analysis using eGFR_crea_, a total of 2,277 Swiss-AF participants were available for sensitivity analysis using eGFR_crea–cys_. Consistent with the main analysis, in a multivariable model adjusting for all clinical variables (Table 2), eGFR_crea–cys_ was strongly associated with sNfL, with a curvilinear relationship including a linear, quadratic, and cubic component (modeled as such based on AIC, Supplementary Table 1). BMI was also strongly associated with sNfL, as was age, diabetes mellitus, and history of stroke or TIA. For all associations, the coefficients were of similar magnitude as in the main analysis. Consistent with the main analysis, there was evidence for an interaction between eGFR_crea–cys_ and age (*p*_*interaction*_ < 0.001). The interaction between BMI and age was even weaker than in the main analysis (*p*_*interaction*_ = 0.057). Examination of the R^2^_adj_ of different models containing age, eGFR_crea–cys_, and BMI either alone or in combination with one another revealed similar results with the main analysis, with eGFR_crea–cys_ explaining a substantial proportion of the sNfL variance beyond that explained by age (Table 3).

A total of 1,402 Swiss-AF patients were available for the sensitivity analysis including MRI data and using eGFR_crea–cys_. As in the main analysis, in the multivariable model including all clinical variables, vascular brain lesions, and nBV, both eGFR_crea–cys_ and BMI remained in the model after backward variable elimination, as did age, its interaction with eGFR_crea–cys_, diabetes mellitus, and history of stroke or TIA (Supplementary Table 3). The R^2^_adj_ of this model was 52% with eGFR_crea–cys_, dropping to 36% after excluding eGFR_crea–cys_.

## Discussion

This cross-sectional study on the association of eGFR and BMI with sNfL concentrations in a large elderly cohort of AF patients showed that both eGFR (estimated using either creatinine or creatinine and cystatin C) and BMI were strongly associated with sNfL concentrations. This was true even after adjustment for other parameters known to contribute to sNfL concentrations, including age, clinical comorbidities, and MRI characteristics. Furthermore, eGFR, but not BMI, conferred a substantial increase in the explanatory power of models predicting sNfL concentrations, which was additional and independent to the contribution of age.

Our finding of a strong negative association of eGFR with sNfL concentrations confirms and refines previous observations (Korley et al., 2019; Akamine et al., 2020). An inverse association between eGFR and blood NfL concentrations was recently shown in smaller samples of elderly patients with diabetes and healthy controls, and the renal clearance of blood NfL was proposed as one potential explanation (Korley et al., 2019; Akamine et al., 2020). Here, we show that this association is independent of age, BMI, and pre-existing disease (diabetes, stroke history, and other cardiovascular comorbidities). The association between eGFR and sNfL was maintained independent of the method used for calculating eGFR, that is, based on creatinine alone or combined creatinine–cystatin C. Importantly, the association persisted even after adjustment for brain volume, as well as for the presence and burden of ischemic infarcts and small vessel disease markers on neuroimaging, which are known to be associated with sNfL concentrations (Gattringer et al., 2017; Duering et al., 2018; Tiedt et al., 2018; Polymeris et al., 2020), indicating that it is not mediated through structural brain pathology. These findings further support that, apart from NfL release from damaged neurons, renal clearance seems to be a predominant factor determining NfL levels. Combined investigations of NfL in cerebrospinal fluid (CSF), blood, and urine to confirm this are now under way in our laboratory. Additionally, we show here that the association of eGFR with sNfL is non-linear, with a steeper slope in lower eGFR values. Taken together with our finding that the association between sNfL and eGFR depends on age (which indicates that the impact of renal impairment on sNfL levels is even more pronounced in older than in younger patients), these data stress the importance of accounting for renal function when evaluating blood NfL concentrations in elderly populations, in whom chronic kidney disease is highly prevalent (Coresh et al., 2003).

We also found a strong inverse association between BMI and sNfL. This is in line with previous observations from large cohorts of young patients with multiple sclerosis and healthy controls, where a larger distribution volume and specifically a larger total blood volume was postulated to be a modifier of blood NfL levels (Manouchehrinia et al., 2020). Here, we expand on these findings by demonstrating that the association holds true also among elderly individuals, and is independent of age, eGFR, cardiovascular comorbidities, and the presence and burden of vascular brain lesions on neuroimaging. Furthermore, we confirmed the linearity of the association and found only a weak interaction with age. These findings strengthen the evidence for the validity of this relationship and for dilution as the underlying mechanism (Barro et al., 2020; Manouchehrinia et al., 2020). It seems therefore appropriate to account for BMI when examining blood NfL concentrations across the entire age spectrum.

Our study provides a comprehensive assessment of the relative contribution of eGFR and BMI in determining NfL serum levels as physiological factors important in its homeostasis in the elderly. After adjustment for age and comorbidities, and regardless of the GFR estimation formula, both eGFR and BMI showed an independent, strong inverse association with sNfL levels, with effect sizes in a similar order of magnitude as age. However, only age and eGFR explained relevant proportions of the sNfL variance. Age alone explained about one-fourth, GFR alone explained approximately one-third, and their combination almost half of the variance of sNfL concentrations in this elderly cardiovascular cohort. Adding BMI did not substantially increase the explanatory power of the model. Taken together, these findings suggest that diagnostic and prognostic applications of sNfL in elderly populations should account not only for age, but also for renal function to increase their clinical meaningfulness, while the contribution of BMI seems to be less important.

Consistent with our findings, two very recent studies also demonstrated the importance of renal function as a contributor to sNfL levels (Koini et al., 2021; Ladang et al., 2022). While these studies featured smaller samples from normal aging cohorts, they further support the key conclusions of our study which examined a significantly larger sample of elderly patients with cardiovascular disease. Consequently, a large reference database for sNfL levels developed recently from data of younger individuals to optimize the use of sNfL for individual application in patients with multiple sclerosis excluded control persons with eGFR < 60 ml/min/1.73 m^2^ (Benkert et al., 2022).

The strengths of this study include: (i) the large sample size of elderly patients with a detailed and standardized clinical and neuroimaging characterization, allowing for the exhaustive adjustment for multiple factors that are known to contribute to sNfL concentrations, indicating that the observed associations are not spurious but reflect true relationships; (ii) the estimation of GFR using two different approaches [the CKD-EPI formula using creatinine alone and the more accurate combined creatinine–cystatin C formula (Inker et al., 2012)] that yielded highly consistent results; and (iii) comprehensive statistical modeling investigating not only the association of eGFR and BMI with sNfL concentrations, but also their relative contribution to the variance of sNfL concentrations.

We acknowledge the following limitations: (i) The study’s cross-sectional design, which allows only for the assessment of association but not causality thereof. (ii) Although our results persisted after adjustment for brain MRI characteristics, we were not able to adjust our analyses for diseases of the peripheral nervous system, which were not systematically collected in Swiss-AF but might contribute to sNfL concentrations (Khalil et al., 2018). (iii) As Swiss-AF included exclusively AF patients, we did not have a comparison group of elderly individuals without this arrhythmia. However, in light of recent studies showing consistent results in other patient populations, this limitation is unlikely to have influenced our key findings. (iv) As the Swiss-AF biosampling protocol did not include the acquisition of CSF or urine, this study was not able to examine whether the observed associations are exclusive to blood concentrations of NfL, and we may only speculate on their underlying mechanisms.

In conclusion, this study represents a comprehensive appraisal of how physiological factors including renal function and BMI are associated with and contribute to blood NfL concentrations in the elderly, thereby providing important insights into the homeostasis of this increasingly used biomarker. The role of renal function and BMI in the prediction of neurological outcomes with sNfL needs to be evaluated in prospective studies.

## Data Availability Statement

The Swiss-AF consent forms, as approved by the ethics committee, do not allow for the data to be made publicly available. Researchers may contact the authors for the potential submission of research proposals for future analyses or independent verification of our results.

## Ethics Statement

Swiss-AF including this study was approved by Ethics Committee of Northwest and Central Switzerland (PB_2016-00793). The patients provided their written informed consent to participate in this study.

## Author Contributions

AP, LB, and JK conceived the study and drafted the manuscript, with additional support from FH, PB, and MC. FH performed the statistical analyses. All authors contributed to study design, data acquisition and analysis, and critically revised the manuscript.

## Conflict of Interest

GM: consultant fees from Astra Zeneca, Bayer, Boehringer-Ingelheim, Novartis, outside of the submitted work. GMDM: Support from SNSF, “Spezialprogramm Nachwuchsförderung Klinische Forschung,” University of Basel, Science Funds (Wissenschaftspool) University Hospital Basel, Swiss Heart Foundation, Bangerter-Rhyner-Stiftung, Swisslife Jubiläumsstiftung for Medical Research, Swiss Neurological Society, Fondazione Dr. Ettore Balli, De Quervain research grant, Thermo Fisher GmbH; Travel honoraria Bayer, BMS/Pfizer; Speaker honoraria Bayer, Medtronic; Steering committee PACIFIC Stroke, Industry payments to the research fund of the University Hospital Basel. SE: Travel or speaker honoraria from Bayer, Boehringer-Ingelheim, Daiichi-Sankyo; Scientific advisory boards Bayer, Boehringer-Ingelheim, BMS/Pfizer, MindMaze; Editorial board of Stroke; Research funding to his institutions from Pfizer, Stago, Daiichi-Sankyo, Science Funds [Wissenschaftsfonds] University Hospital Basel, University Basel, “Wissenschaftsfonds Rehabilitation” University Hospital for Geriatric Medicine Felix Platter, “Freiwillige Akademische Gesellschaft Basel,” Swiss Heart Foundation, SNSF. DC: consulting fees from Roche Diagnostics, outside of the current work. MK: fees from Bayer, Boehringer-Ingelheim, BMS/Pfizer, Daiichi-Sankyo, Medtronic, Biotronik, Boston Scientific, Johnson&Johnson, Roche; grants from Bayer, Pfizer, Boston Scientific, BMS, Biotronik, Daiichi-Sankyo. LB: fees and non-financial support from Amgen, Bayer; fees from BMS, Claret Medical and InnovHeart; grants from AstraZeneca, SNSF, University of Basel, Swiss Heart Foundation, outside the submitted work. JK: speaker fees, research support, travel support, and/or advisory boards Swiss Multiple Sclerosis Society, SNSF, University of Basel, Progressive Multiple Sclerosis Alliance, Bayer, Biogen, Celgene, Merck, Novartis, Octave Bioscience, Roche, Sanofi. The remaining authors declare that the research was conducted in the absence of any commercial or financial relationships that could be construed as a potential conflict of interest.

## Publisher’s Note

All claims expressed in this article are solely those of the authors and do not necessarily represent those of their affiliated organizations, or those of the publisher, the editors and the reviewers. Any product that may be evaluated in this article, or claim that may be made by its manufacturer, is not guaranteed or endorsed by the publisher.
