# Supplementary material for: Renal Function and Body Mass Index Contribute to Serum Neurofilament Light Chain Levels in Elderly Patients With Atrial Fibrillation
Source: Front Neurosci. 2022 Apr 14;16:819010. doi: 10.3389/fnins.2022.819010 (PMC9047720; doi:10.3389/fnins.2022.819010)
Supplement: Supplementary file 1 [file Data_Sheet_1.pdf]

Supplementary Material

Renal function and body mass index contribute to serum neurofilament light chain levels in elderly patients with atrial fibrillation

Polymeris et al.

Supplementary Figure 1. Study flowchart

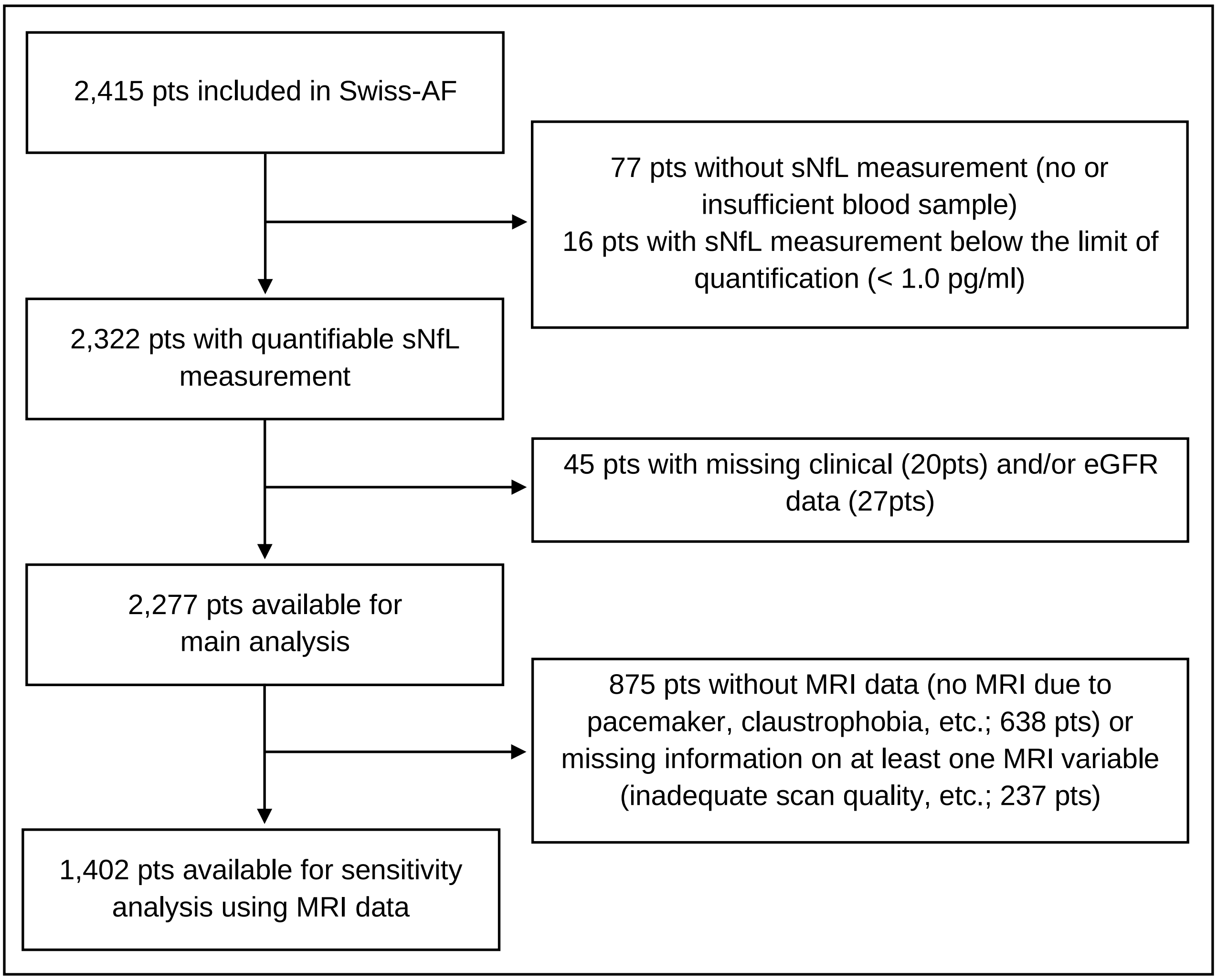

Supplementary Table 1. Linear, quadratic and cubic modelling of the association of eGFR and age with sNfL

**Model** (N= 2,277)	**Component**	**$eGFR_{crea}$** (centered; per10 ml/min/1.73m^2)				**$eGFR_{crea-cys}$** (centered; per10 ml/min/1.73m^2)				**Age** (centered; per decade)			
		**β_{mult}**	**95%-CI**	**p-value**	**AIC**	**β_{mult}**	**95%-CI**	**p-value**	**AIC**	**β_{mult}**	**95%-CI**	**p-value**	**AIC**
**Linear**	linear	0.810	[0.800, 0.820]	<0.001	3702	0.811	[0.802, 0.819]	<0.001	3470	1.497	[1.456, 1.539]	<0.001	3930
**Quadratic**	linear	0.812	[0.802, 0.822]	<0.001	3644	0.807	[0.799, 0.816]	<0.001	3396	1.501	[1.457, 1.546]	<0.001	3932
	quadratic	1.020	[1.015, 1.025]	<0.001		1.018	[1.014, 1.021]	<0.001		1.005	[0.984, 1.027]	0.622	
**Cubic**	linear	0.842	[0.824, 0.860]	<0.001	**3629***	0.827	[0.813, 0.842]	<0.001	**3386***	1.571	[1.504, 1.641]	<0.001	3927
	quadratic	1.019	[1.014, 1.024]	<0.001		1.020	[1.016, 1.024]	<0.001		0.979	[0.952, 1.007]	0.147	
	cubic	0.996	[0.994, 0.998]	<0.001		0.998	[0.997, 0.999]	<0.001		0.978	[0.963, 0.994]	0.006	
**Cubic without quadratic term**	linear	0.848	[0.830, 0.866]	<0.001	3678	0.816	[0.802, 0.830]	<0.001	3471	1.555	[1.492, 1.621]	<0.001	**3927***
	cubic	0.995	[0.993, 0.997]	<0.001		0.999	[0.998, 1.001]	0.333		0.986	[0.974, 0.997]	0.016	

eGFR, estimated glomerular filtration rate; AIC, Akaike's information criterion

*best model, selected based on AIC values and degrees of freedom

Supplementary Table 2. Model-based estimates for the association of $eGFR_{crea}$ and BMI with sNfL in four age-quartile subgroups, describing the changes in curvature ($eGFR_{crea}$) or slope (BMI) in each age subgroup

Variable	**age 37.0 – 68.3 years** (N = 570)			**age 68.3 – 73.6 years** (N = 569)			**age 73.6 – 79.1 years** (N = 569)			**age 79.1 – 95.2 years** (N = 569)		
	β_{mult}	**95%-CI**	**p-value**	β_{mult}	**95%-CI**	**p-value**	β_{mult}	**95%-CI**	**p-value**	β_{mult}	**95%-CI**	**p-value**
$eGFR_{crea}$* (per 10 ml/min/1.73m^2)	0.876	[0.836, 0.919]	<0.001	0.880	[0.839, 0.922]	<0.001	0.902	[0.864, 0.941]	<0.001	0.926	[0.890, 0.965]	<0.001
[$eGFR_{crea}$* (per 10 ml/min/1.73m^2)]^2	1.017	[1.003, 1.031]	0.017	1.021	[1.008, 1.034]	0.001	1.029	[1.015, 1.043]	<0.001	1.021	[0.999, 1.043]	0.067
[$eGFR_{crea}$* (per 10 ml/min/1.73m^2)]^3	0.998	[0.994, 1.002]	0.356	0.995	[0.991, 0.999]	0.022	0.997	[0.993, 1.002]	0.269	0.994	[0.988, 1.000]	0.051
BMI* (per 5 kg/m^2)	0.925	[0.886, 0.966]	<0.001	0.903	[0.863, 0.944]	<0.001	0.872	[0.831, 0.915]	<0.001	0.866	[0.827, 0.906]	<0.001

eGFR, estimated glomerular filtration rate; BMI, body mass index

*centered on its mean; estimates from multivariable models including history of hypertension, diabetes, stroke or transient ischemic attack, peripheral artery disease, heart failure, mean arterial pressure, past smoker, current smoker, alcohol consumption and the interaction $eGFR_{crea}$ x diabetes

Supplementary Table 3. Multivariable model for the association of eGFR and BMI with sNfL including adjustment for MRI variables

**Variables** (N=1,402)	**using $eGFR_{crea}$**		**using $eGFR_{crea-cys}$**	
	**β_{mult}**	**95%-CI**	**β_{mult}**	**95%-CI**
Age (centered; per decade)	1.249	[1.181, 1.320]	1.203	[1.140, 1.269]
$[\text{Age (centered; per decade)}]^3$	0.984	[0.969, 1.000]	0.984	[0.969, 0.999]
eGFR (centered; per 10 ml/min/1.73m^2)	0.889	[0.866, 0.912]	0.878	[0.859, 0.898]
$[\text{eGFR (centered; per 10 ml/min/1.73}m^2)]^2$	1.025	[1.017, 1.034]	1.023	[1.016, 1.029]
$[\text{eGFR (centered; per 10 ml/min/1.73}m^2)]^3$	0.998	[0.995, 1.000]	0.997	[0.996, 0.999]
BMI (centered; per 5 kg/m^2)	0.902	[0.877, 0.928]	0.893	[0.869, 0.917]
History of hypertension	eliminated*		eliminated*	
History of diabetes mellitus	1.172	[1.087, 1.263]	1.168	[1.087, 1.256]
History of stroke or transient ischemic attack	1.078	[1.005, 1.156]	1.073	[1.002, 1.148]
History of peripheral artery disease	eliminated*		eliminated*	
History of heart failure	1.064	[0.999, 1.133]	eliminated*	
Mean arterial pressure (per 1 mmHg)	0.998	[0.996, 1.000]	eliminated*	
Past smoker (ref: non-smoker)	eliminated*		eliminated*	
Current smoker (ref: non-smoker)	eliminated*		eliminated*	
Alcohol consumption (per 1 std.drink/d)	eliminated*		eliminated*	
Presence of SNCIs	eliminated*		eliminated*	
Volume of SNCIs (log-transformed, centered)	eliminated*		eliminated*	
Presence of LNCCIs	1.072	[1.003, 1.145]	1.064	[0.998, 1.134]
Volume of LNCCIs (log-transformed, centered)	1.047	[1.019, 1.076]	1.047	[1.020, 1.074]
Presence of microbleeds	1.076	[1.011, 1.145]	1.068	[1.006, 1.135]
Count of microbleeds	eliminated*		eliminated*	
Volume of WMLs (log-transformed, centered)	1.042	[1.021, 1.065]	1.037	[1.017, 1.059]
Normalized brain volume (per 1 cm^3)	0.9998	[0.9995, 1.0001]	eliminated*	
Interaction eGFR x age	1.021	[0.999, 1.044]	1.023	[1.004, 1.042]
Interaction BMI x age	eliminated*		eliminated*	
Interaction eGFR x diabetes	eliminated*		eliminated*	

BMI, body mass index; eGFR, estimated glomerular filtration rate; SNCIs, small non-cortical infarcts; LNCCIs; large non-cortical or cortical infarcts; WMLs, white matter lesions

*stepwise backward elimination based on Akaike’s information criterion
